# *In situ* removal of carbon contamination from a chromium-coated mirror: ideal optics to suppress higher-order harmonics in the carbon *K*-edge region

**DOI:** 10.1107/S1600577515015040

**Published:** 2015-09-26

**Authors:** Akio Toyoshima, Takashi Kikuchi, Hirokazu Tanaka, Kazuhiko Mase, Kenta Amemiya

**Affiliations:** a Institute of Materials Structure Science, KEK, 1-1 Oho, Tsukuba, Ibaraki 305-0801, Japan; b Department of Materials Structure Science, SOKENDAI (The Graduate University for Advanced Studies), 1-1 Oho, Tsukuba, Ibaraki 305-0801, Japan

**Keywords:** carbon contamination, *in situ* carbon removal, chromium-coated optics, carbon *K*-edge, higher-order harmonics

## Abstract

Carbon-free chromium-coated optics are ideal in the carbon *K*-edge region (280–330 eV) because the reflectivity of first-order light is larger than that of gold-coated optics while the second-order harmonics (560–660 eV) are significantly suppressed by chromium *L*-edge and oxygen *K*-edge absorption. Here, chromium-, gold- and nickel-coated mirrors have been adopted in the vacuum ultraviolet and soft X-ray branch beamline BL-13B at the Photon Factory in Tsukuba, Japan. Carbon contamination on the chromium-coated mirror was almost completely removed by exposure to oxygen at a pressure of 8 × 10^−2^ Pa for 1 h under irradiation of non-monochromated synchrotron radiation. The pressure in the chamber recovered to the order of 10^−7^ Pa within a few hours. The reflectivity of the chromium-coated mirror of the second-order harmonics in the carbon *K*-edge region (560–660 eV) was found to be a factor of 0.1–0.48 smaller than that of the gold-coated mirror.

## Abstract 2

Carbon contamination of a chromium-coated mirror was removed by exposure to oxygen activated with non-monochromated synchrotron radiation. Higher-order harmonics of the chromium-coated mirror are much smaller than those of a gold-coated mirror in the carbon *K*-edge region.

## 1. Introduction   

Carbon contamination of optics is a serious issue in synchrotron radiation (SR) facilities because it decreases the quality of experimental data such as near-edge X-ray absorption fine structure (NEXAFS), resonant photoemission and resonant soft X-ray emission spectra in the carbon *K*-edge region. Recently, we developed an *in situ* method to remove carbon contamination from gold-coated optics in a vacuum ultraviolet and soft X-ray (VSX) undulator beamline (Toyoshima *et al.*, 2012 ▸). Another important issue is to suppress higher-order harmonics in the carbon *K*-edge region. Traditional higher-order suppressors involve the use of either rare gases, thin films or multiple mirror reflections (Samson & Ederer, 2000 ▸). A rare gas cell is an excellent higher-harmonics suppressor in the 6–30 eV region (Suits *et al.*, 1995 ▸), but it requires an efficient differential pumping system. Thin films made of suitable materials such as aluminium, indium, tin, carbon or boron are widely used as band-pass filters in the VSX region (Powell *et al.*, 1990 ▸). Multiple mirror reflections are traditional low-pass filters in the VSX region (Waki *et al.*, 1989 ▸). A disadvantage of the thin-film filters and multiple mirror reflections is that the transmission of the fundamental is not large (typically less than 70%).

To suppress higher-order harmonics in the carbon *K*-edge region, we adopted a chromium-coated mirror. Chromium-coated optics are better than gold-coated optics for the carbon *K*-edge region (280–330 eV) because of the larger reflectivity and because second-order harmonics (560–660 eV) are significantly suppressed by chromium *L*-edge and oxygen *K*-edge absorption. In the present paper we report on an *in situ* method to remove carbon contamination from chromium-coated optics. Carbon contamination on a chromium-coated mirror was almost completely removed by exposure to oxygen at a pressure of 8 × 10^−2^ Pa for 1 h under irradiation of non-monochromated SR. The reflectivity of the chromium-coated mirror was observed to be a factor of 1.3 larger than that of a gold-coated mirror in the carbon *K*-edge region (280–330 eV). On the other hand, the reflectivity of the chromium-coated mirror in the second-order harmonics region of the carbon *K*-edge (560–660 eV) was a factor of 0.1–0.48 smaller than that of the gold-coated mirror.

## 2. Beamline   

The VSX undulator beamline BL-13A at the Photon Factory (PF) has a Monk–Gillieson-type monochromator to cover a wide photon energy range at a high energy resolution (Amemiya & Ohta, 2004 ▸). The details of BL-13A have been described elsewhere (Mase *et al.*, 2010*a* ▸; Toyoshima *et al.*, 2011 ▸, 2013 ▸). BL-13A consists of a focusing pre-mirror (M1), a plane mirror (M2), two varied-line-spacing plane gratings (VLSGs; 300 and 1000 lines mm^−1^), an exit slit and two focusing post-mirrors (M3A, 2 m:2 m focusing; M3A′, 2 m:6 m focusing) (Toyoshima *et al.*, 2011 ▸). The specifications of BL-13A are as follows: photon energy region of 30–1600 eV, photon flux of 10^8^–10^11^ photons s^−1^ and photon energy resolution (*E*/Δ*E*) of 10000 at a photon energy of 401 eV.

M1, M2, the VLSGs, M3 and M3′ are coated with a gold film of thickness 1000 or 500 Å. M1, M2 and the VLSGs are cooled with water at room temperature (Toyoshima *et al.*, 2011 ▸). SR is horizontally reflected by M1, M3 and M3′, and vertically reflected by M2 and the VLSGs. The grazing angle of M1, M3 and M3′ is 2°, and the including angles of the 300 and 1000 lines mm^−1^ VLSGs are 174.9° and 171.3°, respectively, at 290 eV. A planar undulator is used as the source point (Sasaki *et al.*, 1989 ▸) and, unless otherwise stated, the undulator gap is fixed at 154 mm during carbon removal and photon intensity measurements. The first, third and fifth undulator peaks appear at 113, 339 and 564 eV for this gap. The base pressure of every chamber is maintained below 1 × 10^−8^ Pa. Between the front-end and the M1 chamber there are a quadruple-mask chamber, masks, a square duct, movable water-cooling quadruple masks, a photon position monitor and a shutter chamber (Mase *et al.*, 2010*b* ▸; Tanaka *et al.*, 2011 ▸). As these components constitute an efficient differential pumping system, the change in the vacuum level in the storage ring was negligible even when oxygen gas with a pressure of 10^−4^ Pa is supplied in the M1 chamber. Carbon contamination of the optics in BL-13A was removed almost completely by exposing the optics to oxygen gas at a pressure of 10^−1^–10^−4^ Pa for 17–20 h under irradiation of non-monochromated SR (Toyoshima *et al.*, 2012 ▸). Following carbon removal, oxygen gas with a pressure of 10^−7^ Pa is constantly supplied in the M1 and M2/VLSGs chambers to suppress carbon contamination.

**Figure 1:**
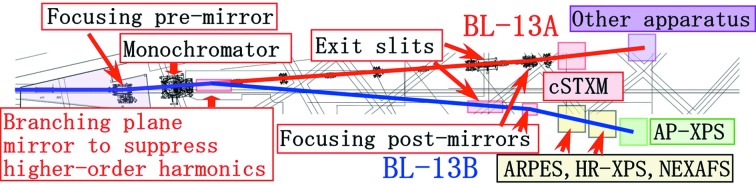
Floor layout of BL-13A and BL-13B at the Photon Factory. AP-XPS denotes ambient pressure X-ray photoelectron spectroscopy, ARPES denotes angle-resolved photoemission spectroscopy and HR-XPS denotes high-resolution X-ray photoelectron spectroscopy.

**Figure 2:**
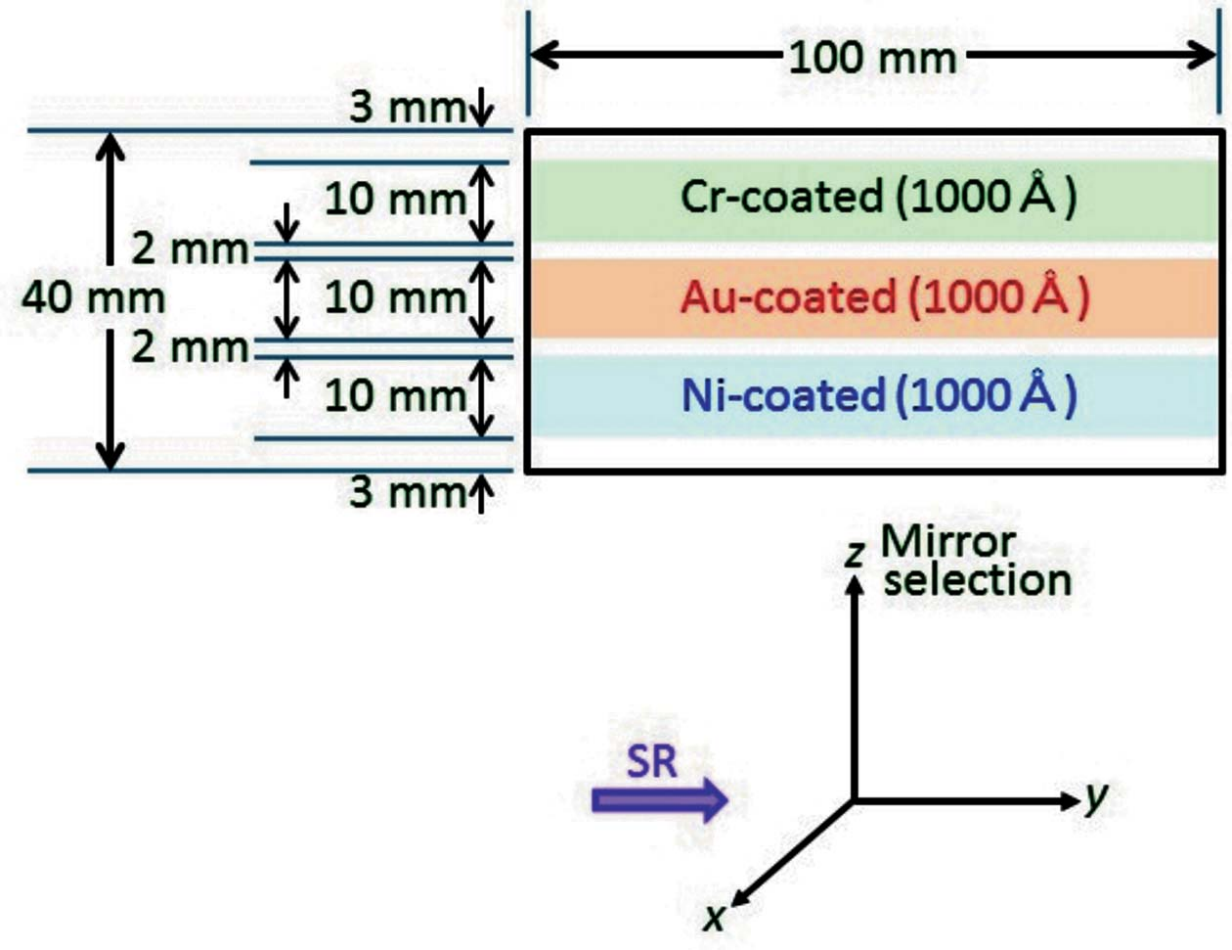
Branching plane mirror (Mp) to suppress higher-order harmonics (upper view) and the definition of the *x*-, *y*- and *z*-axes (lower view). Mp is positioned with its *x*-axis normal to the mirror surface. The *y*-axis is in the direction of the SR. The *z*-axis is in the plane of the mirror surface, perpendicular to the direction of the SR. The chromium-, gold- and nickel-coated mirrors can be easily selected by adjusting the *z*-position of Mp.

**Figure 3:**
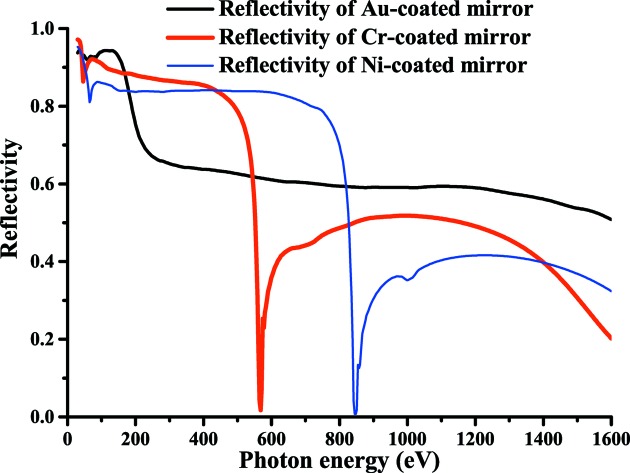
Reflectivity of chromium-, gold- and nickel-coated mirrors for a grazing angle of 2° and *p*-polarization calculated using the Center for X-ray Optics website (http://henke.lbl.gov/optical_constants/mirror2.html).

**Figure 4:**
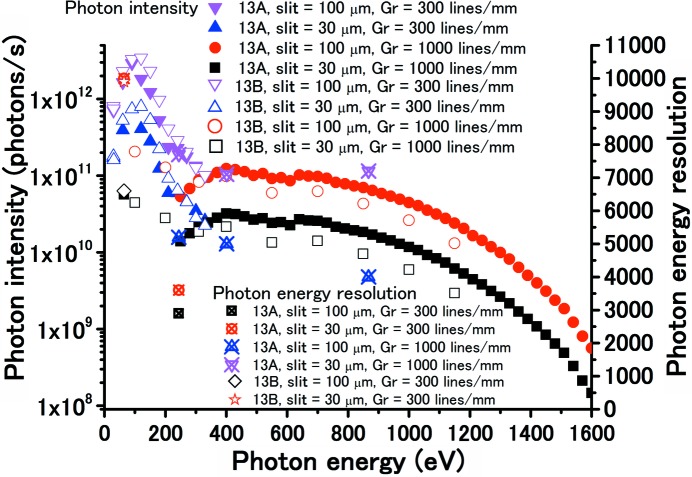
Typical photon intensity and photon energy resolution (*E*/Δ*E*) of BL-13A (Toyoshima *et al.*, 2013 ▸) and 13B in the photon energy region 30–1600 eV.

Recently, we developed a branch VSX beamline for surface chemistry studies (BL-13B, Fig. 1 ▸) and opened it for users in October 2013. A branching plane mirror (Mp) with a grazing angle of 2° is used to reflect SR to BL-13B. BL-13B contains an exit slit, two focusing post-mirrors (M3B, 2 m:2 m focusing; M3B′, 2 m:6 m focusing) and a Si photodiode to measure the photon intensity. The Mp substrate is coated with 1000 Å-thick chromium, gold and nickel layers as shown in Fig. 2 ▸. The chromium-, gold- and nickel-coated mirrors can be easily selected by adjusting the *z*-position of Mp. Chromium-coated optics are ideal in the carbon *K*-edge region (280–330 eV) because of the large reflectivity and suppression of higher-order harmonics (Fig. 3 ▸). In contrast, nickel-coated optics are suitable in the nitrogen *K*-edge region (395–445 eV) because higher-order harmonics are suppressed (Fig. 3 ▸). The Mp and M3B/M3B′ chambers are pumped with oil-free turbomolecular pumps and non-evaporable getter (NEG) pumps. No ion sputtering pumps are used because carbon-contaminated ion sputtering pumps are thought to produce hydrocarbons by collision with ionized residual hydrogen gas (Yamada *et al.*, 1982 ▸). Typical base pressures of the Mp and M3B/M3B′ chambers are 3 × 10^−7^ and 4 × 10^−8^ Pa, respectively. Carbon contamination on the gold-coated Mp, M3B and M3B′ were removed with oxygen activated by non-monochromated SR. Typical photon intensities and energy resolutions (*E/*Δ*E*) of BL-13A/13B are shown in Fig. 4 ▸.

## 3. Results and discussion   

### 3.1. *In situ* carbon removal from chromium-coated mirror   

**Figure 5:**
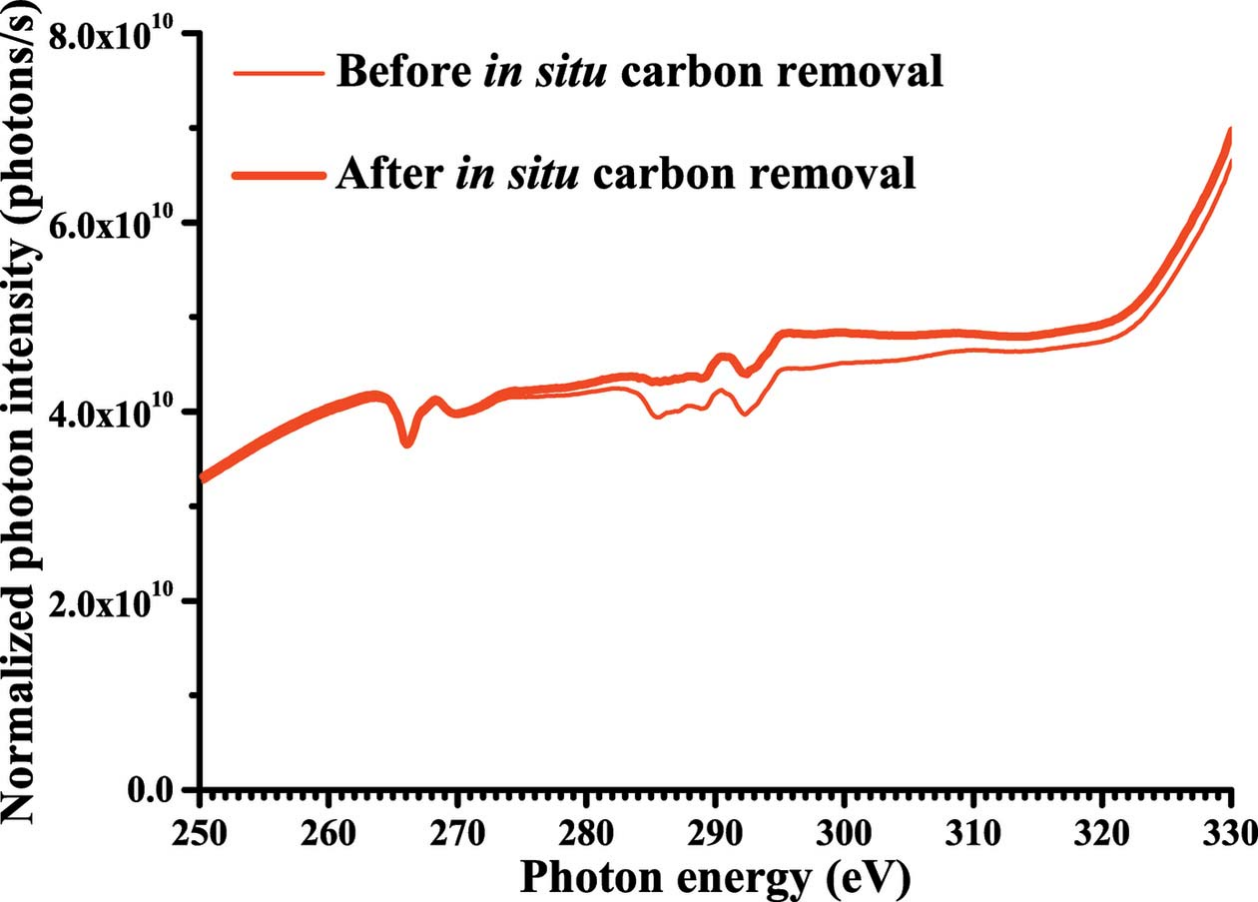
Photon intensity spectra of BL-13B in the carbon *K*-edge region before and after *in situ* carbon removal from the chromium-coated mirror.

Carbon contamination on the chromium-coated mirror was removed by exposure to oxygen at a pressure of 8 × 10^−2^ Pa for 1 h under irradiation of non-monochromated SR. Fig. 5 ▸ shows photon intensity spectra of BL-13B in the carbon *K*-edge region before and after *in situ* carbon removal from the chromium-coated mirror. On the basis of the NEXAFS spectra of graphite (Rosenberg *et al.*, 1986 ▸), we ascribed the small negative peak at 285.5 eV in the spectra obtained before the carbon removal to the C 1*s* → π* transition of graphite-like carbon with a flat-on configuration on the chromium-coated mirror. After the carbon removal, the negative peak at 285.5 eV disappeared and the photon intensity in the carbon *K*-edge region (280–330 eV) increased. These results indicate that the carbon on the chromium-coated mirror has been almost completely removed. Since the photon intensity involves higher-order harmonics, the negative peaks at photon energies of 266, 288 and 292 eV are ascribed to oxygen *K*-edge, chromium *L*_III_-edge and chromium *L*_II_-edge absorption at 532, 576 and 584 eV, respectively.

**Figure 6:**
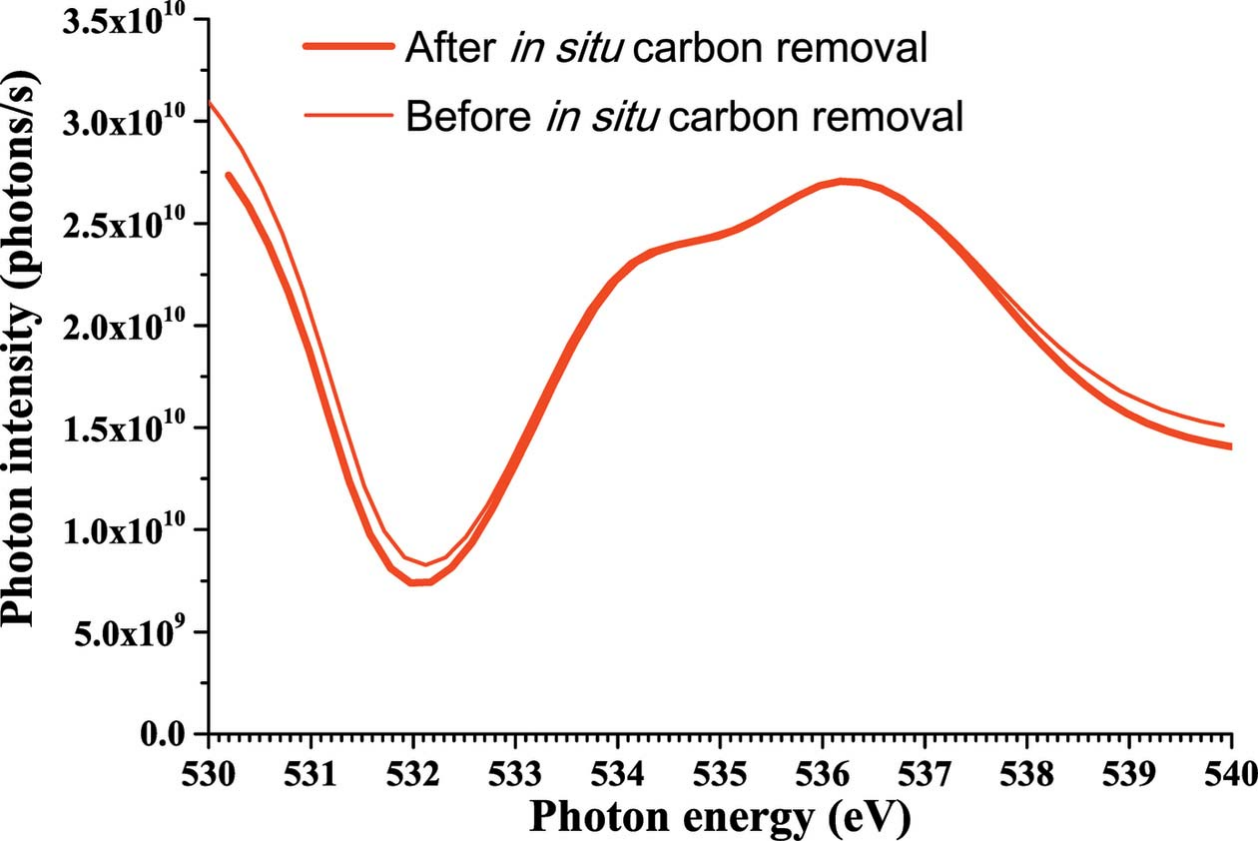
Photon intensity spectra of BL-13B in the oxygen *K*-edge region before and after *in situ* carbon removal from the chromium-coated mirror.

Fig. 6 ▸ shows photon intensity spectra in the oxygen *K*-edge region before and after the carbon removal. The oxygen *K*-edge absorption peak at 532 eV increases slightly after the carbon removal, suggesting that further oxidation of the chromium-coated mirror occurs. Further oxidation of the nickel-coated mirror is thought to be negligible because it is not irradiated by non-monochromated synchrotron radiation when oxygen gas is introduced. The pressure in the chamber recovered to the order of 10^−7^ Pa within a few hours without baking. The beamline can be used without additional commissioning.

### 3.2. Comparison of chromium- and gold-coated mirrors   

**Figure 7:**
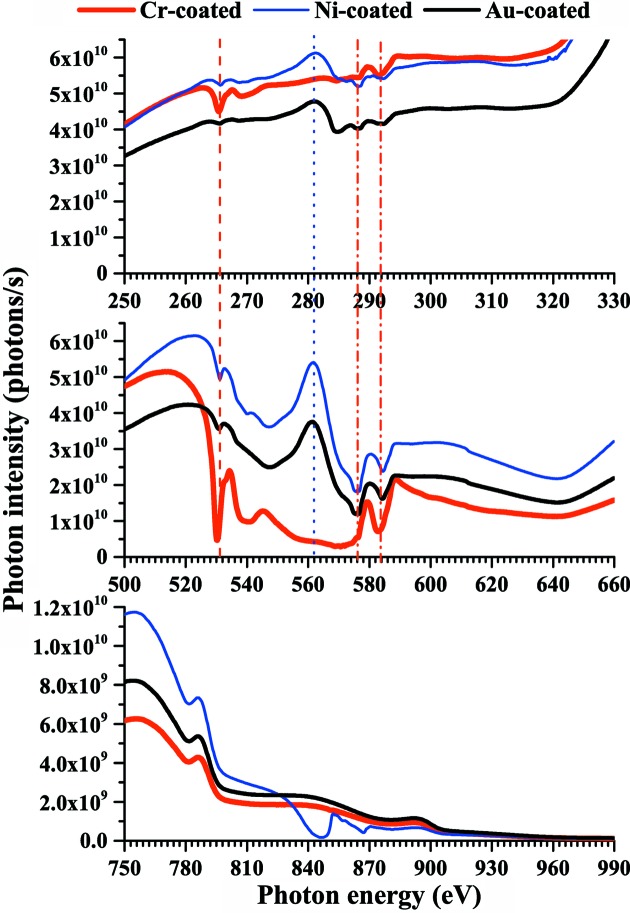
Photon intensity spectra measured with a chromium-, gold-, or nickel-coated mirror in the photon energy regions 250–330 (upper), 500–660 (middle) and 750–990 eV (lower). Higher-order harmonics are involved.

Fig. 7 ▸ shows photon intensity spectra measured with a chromium-, gold- or nickel-coated mirror in the photon energy regions 250–330, 500–660 and 750–990 eV. The chromium *L*_III_-edge and *L*_II_-edge absorption at 576 and 584 eV, respectively, are also observed in the spectra measured with the gold- and nickel-coated mirrors (Fig. 7 ▸, dot-dashed lines) because chromium is used to improve the adhesion property between the substrate and the coating. Oxidized chromium seems to be responsible for the oxygen *K*-edge absorption at a photon energy of 532 eV observed in the spectra measured with the gold- and nickel-coated mirrors (Fig. 7 ▸, dashed line). Since the photon intensity involves higher-order harmonics, the peak at a photon energy of 282 eV in the spectra measured with the nickel- and gold-coated mirrors is assigned to the fifth-order undulator peak at 564 eV (Fig. 7 ▸, dotted line). The peaks at 282 and 564 eV are negligible in the spectra measured with the chromium-coated mirror, suggesting that 564 eV photons are mostly absorbed by oxidized chromium. The photon intensity in the third-order harmonics region of the carbon *K*-edge (840–990 eV) is one order of magnitude smaller than that in the second-order harmonics region (560–660 eV) because the grazing angle is 2°.

**Figure 8:**
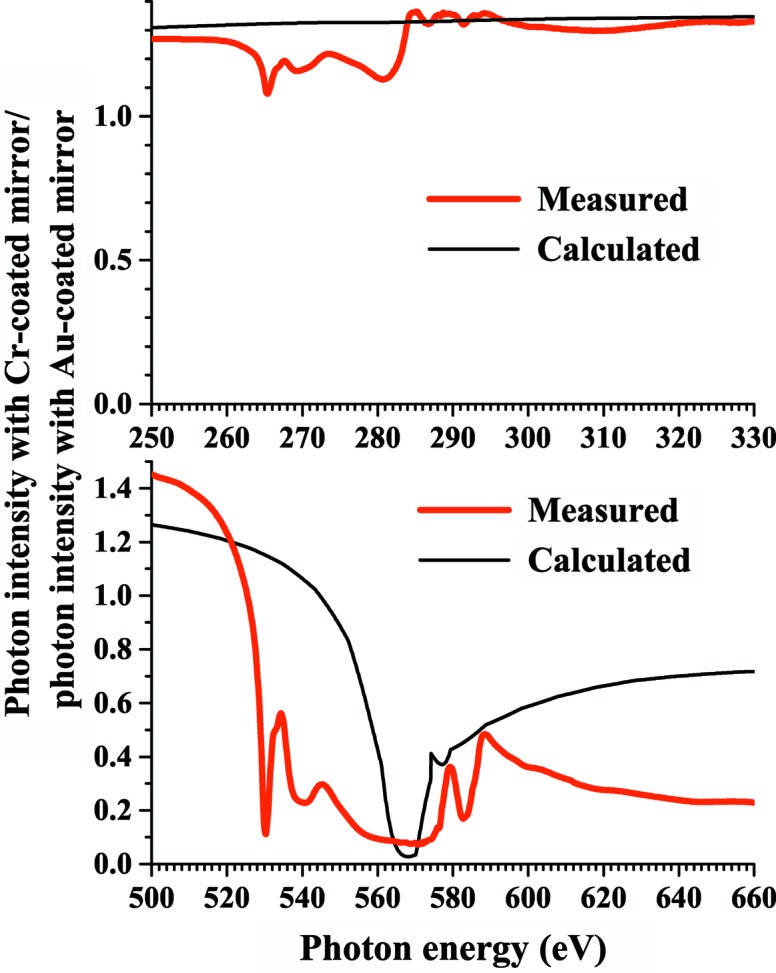
Ratio of measured and calculated photon intensities with a chromium-coated mirror to those with a gold-coated mirror. Data in Figs. 3 ▸ and 7 ▸ are used. The measured ratio is different from the calculated ratio because the surface chromium is oxidized and because the measured photon intensity involves higher-order harmonics.

Fig. 8 ▸ shows the ratio of the photon intensity with the chromium-coated mirror to that with the gold-coated mirror. The measured ratio differs from the calculated ratio because the surface chromium is oxidized and because the measured photon intensity involves higher-order harmonics. The photon intensity with the chromium-coated mirror is a factor of 1.3 larger than that of the gold-coated mirror in the carbon *K*-edge region (280–330 eV), while the former is a factor of 0.1–0.48 smaller than that of the latter in the second-order harmonics region (560–660 eV). These results demonstrate that chromium-coated optics are more advantageous than gold-coated optics in the carbon *K*-edge region.

## 4. Conclusions   

We constructed the branch VSX undulator beamline BL-13B at the PF and opened it for users in October 2013. Plane mirrors coated with gold, chromium or nickel are used to branch SR. Carbon contamination on the chromium-coated mirror was removed by exposure to oxygen at a pressure of 8 × 10^−2^ Pa for 1 h under irradiation of non-monochromated SR. The base pressure of the beamline recovered to 10^−7^ Pa in a few hours without baking. The beamline can be used without additional commissioning. The photon intensity with the chromium-coated mirror is a factor of 1.3 larger than that of the gold-coated mirror in the carbon *K*-edge region (280–330 eV), while the former is a factor of 0.1–0.48 smaller than the latter in the second-order harmonics region (560–660 eV). These results demonstrate that chromium-coated optics are ideal for experiments in which intense SR without higher-order harmonics is required in the carbon *K*-edge region.
